# Development, Characterization and Application of Monoclonal Antibodies against Brazilian Dengue Virus Isolates

**DOI:** 10.1371/journal.pone.0110620

**Published:** 2014-11-20

**Authors:** Camila Zanluca, Giovanny Augusto Camacho Antevere Mazzarotto, Juliano Bordignon, Claudia Nunes Duarte dos Santos

**Affiliations:** 1 Laboratório de Virologia Molecular, Instituto Carlos Chagas (ICC/Fiocruz/PR), Curitiba, Paraná, Brasil; 2 Programa de Pós-Graduação em Biologia Celular e Molecular, Universidade Federal do Paraná (UFPR), Curitiba, Paraná, Brasil; New York State Dept. Health, United States of America

## Abstract

Dengue is the most prevalent human arboviral disease. The morbidity related to dengue infection supports the need for an early, quick and effective diagnostic test. Brazil is a hotspot for dengue, but no serological diagnostic test has been produced using Brazilian dengue virus isolates. This study aims to improve the development of immunodiagnostic methods for dengue virus (DENV) detection through the production and characterization of 22 monoclonal antibodies (mAbs) against Brazilian isolates of DENV-1, -2 and -3. The mAbs include IgG2bκ, IgG2aκ and IgG1κ isotypes, and most were raised against the envelope or the pre-membrane proteins of DENV. When the antibodies were tested against the four DENV serotypes, different reactivity patterns were identified: group-specific, subcomplex specific (DENV-1, -3 and -4 and DENV-2 and -3) and dengue serotype-specific (DENV-2 or -3). Additionally, some mAbs cross-reacted with yellow fever virus (YFV), West Nile virus (WNV) and Saint Louis encephalitis virus (SLEV). None of the mAbs recognized the alphavirus Venezuelan equine encephalitis virus (VEEV). Furthermore, mAbs D3 424/8G, D1 606/A12/B9 and D1 695/12C/2H were used to develop a capture enzyme-linked immunosorbent assay (ELISA) for anti-dengue IgM detection in sera from patients with acute dengue. To our knowledge, these are the first monoclonal antibodies raised against Brazilian DENV isolates, and they may be of special interest in the development of diagnostic assays, as well as for basic research.

## Introduction

Dengue is one of the most prevalent arboviral diseases in tropical and subtropical regions of the world. Over 40% of the world’s population lives in areas at risk of transmission, and there are an estimated 390 million dengue infections each year, of which 96 million manifest disease symptoms [1]. Additionally, it is believed that ∼500,000 cases result in severe disease and ∼12,500 in death each year [2], [3].

Dengue virus (DENV), the causative agent of dengue, is a positive-sense single-stranded RNA virus that belongs to the genus *Flavivirus*, family *Flaviviridae*. The virus is transmitted by *Aedes* (*Stegomyia*) mosquitoes and is classified into four antigenically distinct but closely related serotypes (DENV-1 to -4) [4]. All four DENV serotypes manifest in a wide spectrum of clinical presentations, including severe (hemorrhagic fever, DHF; or shock syndrome, DSS) and non-severe diseases (dengue fever, DF) [5]. DENV infection symptoms are not sufficiently specific to allow clinical differentiation from other acute febrile illnesses, especially in areas where multiple tropical diseases such as malaria, yellow fever, West Nile disease and Saint Louis encephalitis are endemic [6]. There are several dengue vaccine candidates under development, but none is licensed and available [7]. Additionally, there is no specific treatment for dengue, and the most effective protective measures are those that lower the risk of mosquito bites. Thus, early diagnosis is crucial to reducing morbidity and mortality from DHF and DSS.

Laboratory diagnosis of dengue is based on viral isolation in cell culture, reverse-transcriptase/polymerase-chain reaction (RT-PCR) and serological assays [8], [9], [10], [11]. Several immunoassays for DENV, such as enzyme immunoassays, immunochromatographic and dot-blot assays, are commercially available [10], [12], [13], [14], [15]. The IgM antibody capture enzyme-linked immunosorbent assay (MAC-ELISA) is the assay of choice for the serological diagnosis of primary dengue-virus infection [11]. Combined with IgG titers, this assay allows the diagnosis of secondary dengue infection. Furthermore, both IgM and IgG dengue ELISAs are useful tools for seroepidemiological dengue surveillance and can be applied in studies of DENV pathogenesis and host-pathogen relationships [16], [17].

Antibodies have been used in recent decades to diagnose several viral diseases and in investigations of viral structure [18], [19], [20], [21], [22]; however, the heterogeneity of the polyclonal antibodies used in tests can lead to problems in the interpretation, reproducibility and standardization of the assays. To overcome these limitations, several monoclonal antibodies (mAbs) able to bind to specific antigens have been developed [20], [23], [24], [25]. The first serotype-specific mAbs against DENV were developed by Dittmar et al. (1980) [26]. Monoclonal antibodies against DENV have been successfully used for the identification of viral serotypes, flavivirus differentiation and epidemiological studies, as well as for dengue diagnosis and immunotherapy studies [10], [27], [28], [29], [30], [31], [32], [33], [34], [35].

This study reports the development and characterization of twenty-two mAbs against Brazilian DENV isolates. From this panel, three mAbs were tested in an IgM capture assay for the detection of acute dengue patients in Brazil. The monoclonal antibodies generated were group-specific, subcomplex-specific and serotype-specific, representing essential tools for dengue- and serotype-specific diagnosis. Thus, these antibodies have the potential to increase the specificity and sensitivity of dengue diagnosis in Brazil and throughout South America.

## Animals and Methods

### Cell lines and viruses

The mouse myeloma cell line P3x63Ag8.653 (kindly supplied by Dr. Carlos R. Zanetti, from Laboratório de Imunologia Aplicada, at Universidade Federal de Santa Catarina, Florianópolis, Brazil; ATCC CRL-1580) and hybridomas were maintained in RPMI-1640 medium (Cultilab, Campinas, Brazil) supplemented with 20% fetal bovine serum (FBS–Gibco, Grand Island, USA), 23.8 mM sodium bicarbonate, 2.0 mM L-glutamine, 1.0 mM sodium pyruvate, 9.6 mM HEPES and antibiotics (100 IU/ml penicillin, 100 µg/ml streptomycin and 0.25 µg/ml amphotericin B – Sigma-Aldrich, Steinheim, Germany) at 37°C in a 5% CO_2_ atmosphere. C6/36 *Aedes albopictus* cells (ATCC CRL-1660) were cultured in Leibovitz’s L15 medium (Gibco) with 5% FBS, 25 µg/ml gentamicin (Gibco) and 0.27% tryptose at 28°C. Human-derived hepatoma cells (Huh7.5) (ATCC PTA-8561) and Vero E6 cells (Sigma, 85020206) were maintained in Dulbecco’s Modified Eagle Medium/Nutrient Ham F12 (DMEM F12 – Gibco) with 10% FBS, 14.0 mM sodium bicarbonate and antibiotics (100 IU/ml penicillin, 100 µg/ml streptomycin) at 37°C in a 5% CO_2_ atmosphere.

The serotypes DENV-1 (BR/01-MR and BR/90), -2 (BR/01-01 and ICC 266), -3 (290-02) and -4 (TVP 360) were used in this study. DENV-4 TVP 360 is a World Health Organization reference strain, kindly supplied by Dr. Ricardo Galler from Fundação Oswaldo Cruz, Rio de Janeiro, Brazil. DENV-1 BR/01-MR (GenBank AF513110.1) and BR/90 (GenBank AF226685.2); DENV-2 BR/01-01 (GenBank JX073928) and ICC 266 (not sequenced); and DENV3 290-02 (GenBank EF629369.1) are clinical isolates from dengue fever obtained in Brazil between 1990 and 2004. All viruses were amplified and titrated by the foci-forming assay in C6/36 cells [36]. The yellow fever virus (YFV) 17DD vaccine strain (BioManguinhos, Fiocruz, Brazil) was obtained after three passages and titration in Vero cells [37]. The Saint Louis encephalitis virus (SLEV) 78V6507 strain, isolated from *Culex pipiens quinquefasciatus* mosquitoes from Santa Fé Province, Argentina [38]; West Nile virus (WNV) E/7229/06, isolated from a dead horse from Buenos Aires Province, Argentina [39]; and Venezuelan equine encephalitis virus (VEEV) TC38 vaccine strain [40] were kindly supplied by Dr. Marta S. Contiginani from Instituto de Virología Dr. J.M. Vanella, Facultad de Ciencias Médicas, Universidad Nacional de Córdoba.

### Animals and immunization protocol

Ethics statements for all animal procedures were approved by the Ethical Committee on Animal Research of the Universidade Federal do Paraná under the protocol no. 23075.031314/2008-41. Four young adult (30- to 45-day-old) BALB/c mice were used in the immunization protocols for each DENV serotype. All animals were maintained at the Animal Facility of the Instituto Carlos Chagas – FIOCRUZ/PR with water and food *ad libitum* and a light-dark cycle of 12 h/12 h.

Animals were bled by caudal puncture for extraction of pre-immune serum and then immunized with five doses of 1×10^6^ ffu_C6/36_/dose/animal of DENV-1 (BR-01/MR), -2 (BR/01-01) or -3 (BR 290-02). Doses were administered via the intraperitoneal (doses 1 and 3), intradermal (doses 2 and 4) or intravenous route (dose 5), with 1-week intervals between doses. Complete Freund’s adjuvant was used in dose 1 (Sigma-Aldrich), and Alu-Gel-S was used in doses 2 to 4 (Serva, Heidelberg, Germany). No adjuvant was used in the fifth dose.

### Production of monoclonal antibodies

Three days after the final immunization, the mice were anesthetized with ketamine/xylazine (100 and 10 mg/kg, respectively) via the intraperitoneal route and bled by cardiac puncture to obtain post-immune sera. After post-immune sera were obtained, the animals were euthanized by cervical dislocation. Their spleens were removed aseptically, and splenocytes were fused with P3x63Ag8.653 cells using polyethylene glycol (MW 3000–3700; Sigma-Aldrich), as previously described [20]. Hybrid cells were selected by growth in RPMI-1640 (as described above) plus 100 µM hypoxanthine, 0.4 µM aminopterine and 16 µM thymidine (HAT medium–Sigma-Aldrich) for 14 days. The hybridoma supernatants were screened by indirect immunofluorescence assay (IFA), as described below. Hybridomas whose supernatants showed positive results on IFA were stabilized by two successive freeze-thaw cycles. Cells that remained positive after two cycles were subjected to two rounds of the limiting dilution method and stored in liquid nitrogen. The immunoglobulin isotypes of the mAbs were determined using the SBA Clonotyping System/HRP (Southern Biotech, Birmingham, USA), following the manufacturer’s instructions.

### mAb screening

Hybridomas secreting antibodies against DENV were selected by IFA on DENV-infected C6/36 cells and on control uninfected C6/36 cells (MOCK). C6/36 cells (1.0×10^5^ cells/well in 96-well plates) were infected with the corresponding DENV isolate at a multiplicity of infection (MOI) of 1. Cells were fixed 72 h post-infection with methanol:acetone (1∶1 v/v) for at least 30 min at –20°C. Hybridoma supernatants (100 µL) containing the first antibodies were added and incubated for 30 min at 37°C. To detect reactive antibodies, the infected cells were incubated for 1 h at 37°C with Alexa Fluor 488-conjugated anti-mouse immunoglobulins (Sigma–Aldrich). Cell nuclei were labeled with 300 nM of 4′,6-diamidino-2-phenylindole (DAPI) for 5 minutes, followed by 3 washes with 1x PBS. The flavivirus-specific mAb 4G2 (hybridoma D1-4G2-4-15, ATCC HB-112) and a non-correlated mAb that recognizes hantavirus nucleoprotein (clone 572/7A) [20] were used as positive and negative controls, respectively. The immunofluorescence images were captured with a Leica AF6000 Modular System.

### Specificity of anti-dengue virus mAbs

To investigate the specificity of mAbs to DENV proteins, mAbs were used in western blot (WB) assays with the corresponding DENV serotypes. Dengue viruses were obtained from the supernatant of C6/36 cells infected with a MOI of 0.01. Each virus serotype was concentrated by polyethylene glycol precipitation using PEG 8000 at a final concentration of 7%, and purified by sedimentation through a 30%/60% sucrose (in TNE – 20 mM Tris pH 8.0, 150 mM NaCl, 2 mM EDTA) cushion. Further, purified DENV were inactivated by gamma irradiation.

Viral proteins had previously been quantified with the Micro BCA Protein Assay kit (Pierce, Rockford, USA). Three micrograms of purified gamma-irradiated DENV-1 BR/01-MR or DENV-2 BR/01-01, or 12 µg of DENV-3 290-02, were mixed with Laemmli sample buffer, boiled for 3 min and loaded into 13% SDS-PAGE gels [41]. Viral proteins were transferred to nitrocellulose membranes (GE-Healthcare, Little Chalfont, UK). Membranes were incubated first with 5% non-fat milk in TBS-T (20 mM Tris, 137 mM NaCl, pH 7.6, containing 0.05% Tween 20) and then with hybridoma supernatants. Monoclonal antibodies 4G2 and anti-hantavirus 572/7A were used as positive and negative controls, respectively. Anti-mouse IgG conjugated to alkaline phosphatase (1∶7,500; Sigma-Aldrich) was used as a secondary antibody. All incubation steps were conducted for 1 h at room temperature. The reaction was developed using a solution of NBT (nitroblue tetrazolium) and BCIP (5-bromo-4-chloro-3-indolyl-phosphate) (Promega, Madison, USA). mAbs produced against the DENV-2 isolate were also tested by WB with a recombinant domain III peptide from DENV-2 envelope protein (∼12 kDa) expressed in a prokaryotic system. Furthermore, mAbs produced against DENV-3 were tested by IFA with a truncated recombinant E protein from DENV-3 (DENV-3 E Δ_101_) expressed by transfected *Drosophila* S2 cells. S2 cells transfected with the plasmid pMt/Bip/V5-HisA containing the gene of the E protein from DENV-3 strain BR 290-02 (GenBank EF629369.1), deleted from the carboxi-terminal anchor (corresponding to the last 101 amino acids), were cultured in Schneider’s medium (Gibco) with 10% FBS and 25 µg/mL of gentamicin (Gibco). Envelope protein expression was induced by 500 µM of CuSO_4_ for 48 h. After protein induction 1×10^5^ cells/well were added to a 96-well plate. After adhesion, cells were fixed with methanol:acetone, and IFA was performed as described above.

### Reactivity of mAbs against the four DENV serotypes and other flaviviruses and alphaviruses

The reactivity of the mAbs against the four serotypes of DENV was determined using the IFA. C6/36 cells were infected with the DENV-1 (BR/90), -2 (ICC 266), -3 (290-02) or -4 (TVP 360) isolates at a MOI of 1. After 72 h of infection, the cells were fixed in methanol:acetone and assayed by IFA, as previously described. The reactivity of each mAb against the Huh7.5 cells infected with the YFV 17DD strain, the SLEV 78V6507 isolate and the VEEV TC83 strain and Vero E6 cells infected with the WNV E/7229/06 isolate at MOIs of 1 (2.0×10^4^ cells/well) was also assayed by IFA after 72 h, as previously described.

### Conjugation of mAb to horseradish peroxidase (HRP) and application to the development of a capture ELISA

The antibodies were coupled with horseradish peroxidase (HRP) according to a modified periodate procedure [42]. Briefly, mAbs D3 424/8G, D1 606/A12/B9 and D1 695/12C/2H were purified on a protein-G column (GE-Healthcare) according to the manufacturer’s instructions. HRP was structurally modified by sodium periodate and dialyzed against sodium-acetate buffer (pH 4.4) over 16 h at 4°C. The purified mAb diluted in sodium carbonate was added to the HRP solution and mixed for 2 h at room temperature, followed by the addition of a sodium borohydride solution. After 2 h, conjugated antibodies were purified by ammonium sulfate precipitation [43]. The performance of the mAbs D3 424/8G-HRP, D1 606/A12/B9-HRP and D1 695/12C/2H-HRP conjugate was evaluated by an in-house MAC-ELISA using gamma-irradiated purified DENV particles. A MAC-ELISA was performed as described by Takasaki et al. (2002) [44], with minor modifications. A total of twenty-two human serum samples from patients with dengue fever and twenty-four dengue-negative human sera kindly supplied by State Central Laboratory LACEN/PR were tested (Fiocruz Research Ethics Committee under protocol 617-11). A dengue IgM capture ELISA from PanBio (PanBio, Queensland, Australia) was used to diagnose samples for comparison with the results of the in-house assay.

## Results

The fusion experiments (one for DENV-1, one for DENV-2 and another for DENV-3) generated a total of 1,100 hybridomas, which were screened by IFA to evaluate the presence of anti-DENV antibodies. One hundred forty-seven hybridomas (13.4%) were positive for antibody secretion against the corresponding DENV isolate, with different fluorescence levels. The clones were stabilized through two freeze-thaw cycles, resulting in 22 stable hybridomas. Three of these hybridomas produced antibodies against DENV-1 BR/01-MR; three produced antibodies against DENV-2 BR/01-01; and sixteen produced antibodies against DENV-3 BR 290-02. Antibody isotyping revealed ten IgG2b mAbs, seven IgG2a and five IgG1, all possessing kappa light chains (Table 1).

**Table 1:** Monoclonal antibody (mAb) designation and characterization.

mAb	DENV serotype for immunization	Isotype	Reactivity against				Virion protein
			DENV 1	DENV 2	DENV 3	DENV 4	
**D1 463/G6/H2**	DENV 1	IgG1κ	+	−	+	+	E
**D1 695/12C/2H**	DENV 1	IgG1κ	+	+	+	+	E
**D1 606/A12/B9**	DENV 1	IgG1κ	+	+	+	+	prM
**D2 646/9G**	DENV 2	IgG2aκ	−	+	−	−	N.D.
**D2 658/9A**	DENV 2	IgG2aκ	−	+	−	−	N.D.
**D2 332/2D**	DENV 2	IgG2aκ	−	+	−	−	E
**D3 342/5G/G8**	DENV 3	IgG2aκ	−	−	+	−	E
**D3 388/4A/G6**	DENV 3	IgG1κ	−	−	+	−	E
**D3 444/4G/H3**	DENV 3	IgG2bκ	−	−	+	−	E
**D3 389/F4/H10**	DENV 3	IgG1κ	−	−	+	−	E
**D3 441/D1/H2**	DENV 3	IgG2bκ	−	−	+	−	E
**D3 290/4C/G9**	DENV 3	IgG2aκ	−	−	+	−	E
**D3 341/H9/F10**	DENV 3	IgG2aκ	−	−	+	−	E
**D3 344/H1**	DENV 3	IgG2bκ	−	−	+	−	E*
**D3 442/4E/G8**	DENV 3	IgG2bκ	−	−	+	−	E
**D3 242/F1/H2**	DENV 3	IgG2aκ	−	+	+	−	E
**D3 424/8G**	DENV 3	IgG2bκ	+	+	+	+	E
**D3 863/G7/H7**	DENV 3	IgG2bκ	+	+	+	+	prM
**D3 457/H7/H2**	DENV 3	IgG2bκ	+	+	+	+	prM
**D3 443/H12/H6**	DENV 3	IgG2bκ	+	+	+	+	prM
**D3 868/G7/H10**	DENV 3	IgG2bκ	+	+	+	+	prM
**D3 63/F2/G7**	DENV 3	IgG2bκ	+	+	+	+	E**

N.D. not determined; − negative; + positive; E, envelope protein; prM, pre-membrane protein;

*Did not react in the western blot, but recognize recombinant DENV-3 E Δ_101_ protein expressed on *Drosophila* S2 cells on IFA.

**Reacted in the western blot, but did not recognize recombinant DENV-3 E Δ_101_ expressed on S2 cells on IFA.

**Figure 1:**
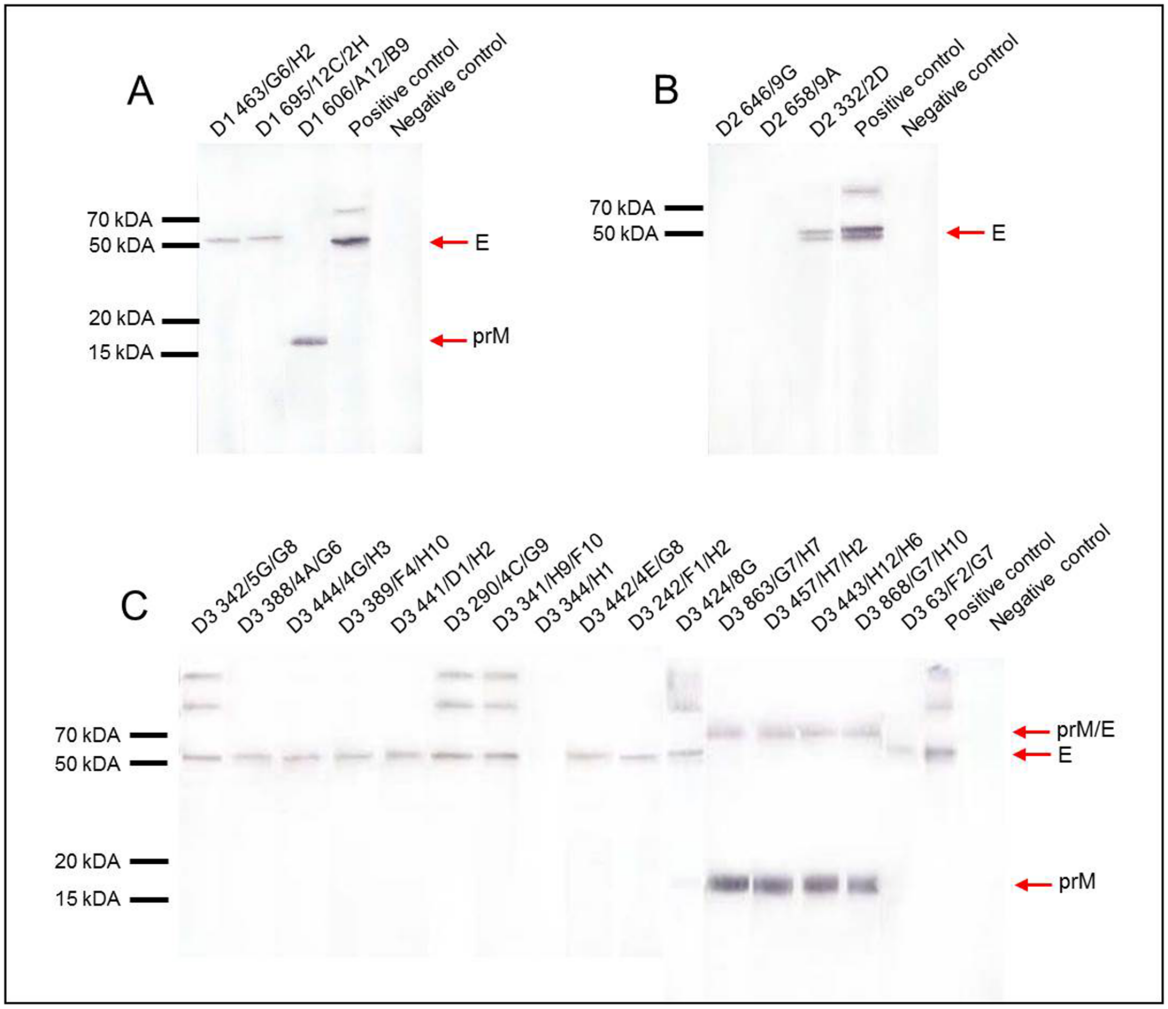
Western blot analysis of mAbs raised against the homologous DENV serotype. Purified gamma-irradiated DENV-1 BR/01-MR (A), DENV-2 BR/01-01 (B), and DENV-3 290-02 (C) were subjected to 13% SDS-PAGE and electroblotted onto nitrocellulose membranes. Proteins were stained with the mAbs, followed by anti-mouse IgG conjugated to alkaline phosphatase. The flavivirus-specific mAb 4G2 and a non-correlated mAb that binds to hantavirus nucleoprotein (clone 572/7A) were used as positive (+) and negative (−) controls, respectively.

Western blot analysis with purified DENV particles showed that fourteen mAbs recognized the envelope protein (E) and five recognized the pre-membrane protein (prM; Figure 1; Figure S1). Additionally, mAbs D2 646/9G, D2 658/9A, and D3 344/H1 showed no reaction to the viral structural proteins on western blot assays (Figure 1). Monoclonal antibodies against DENV-2 were also tested against a recombinant peptide from domain III of the DENV-2 E protein. D2 332/2D reacted specifically to domain III of the E protein while D2 646/9G and D2 658/9A did not (Figure S1). Additionally, on IFA, mAb D3 344/H1 bound the recombinant E protein of DENV-3 expressed on *Drosophila* S2 cells, suggesting that it is directed to a conformational epitope of the E protein (Figure S2).

Interestingly, mAb D3 63/F2/G7 recognized the E protein in the western blot but not in the IFA against recombinant DENV-3 E Δ_101_ protein. From twelve mAbs that reacted against the E protein of DENV-3 by WB only D3 63/F2/G7 does not recognize E protein expressed on *Drosophila* S2 cells, suggesting that this mAb recognizes an epitope located on the carboxi-terminal of the E protein or alternatively, different epitopes conformations are available in the antigens preparations (Figure S2 and Table S1). The positive control 4G2 recognized the E protein in IFA and western blots. No reaction was observed to the anti-hantavirus mAb, which was used in both assays as a negative control (Figure 1; Figure S2).

**Figure 2:**
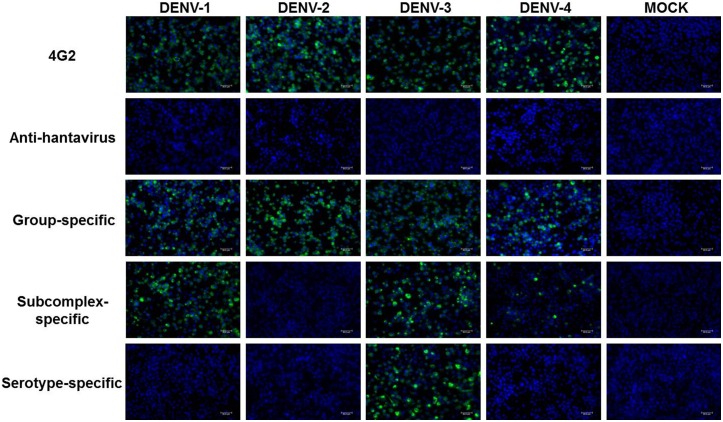
Representation of the reactivities of major groups of monoclonal antibodies. Indirect immunofluorescence of C6/36 cells uninfected (MOCK) or infected with DENV-1 (BR/90), DENV-2 (ICC 266), DENV-3 (290-02) and DENV-4 (TVP 360) isolates. Cells were fixed in methanol:acetone and stained with different mAbs, followed by Alexa-Fluor 488-conjugated anti-mouse immunoglobulin. Monoclonal antibody 4G2 and a non-correlated anti-hantavirus mAb (clone 572/7A) were used as positive and negative controls, respectively. Distinct groups of mAbs were raised against DENV: 1) group-specific (D3 424/8G); 2) subcomplex-specific (Anti-DENV-1, anti-DENV-3 and anti-DENV-4; clone D1 463/G6/H2); and 3) serotype-specific (anti-DENV-3 D3 290/4C/G9) mAbs. Images were produced in a Leica AF6000 Modular System. Scale bars are 30 µm.

To investigate whether the mAbs could be used for diagnostic and epidemiological purposes, the mAbs were assessed for specificity to the different DENV serotypes and to other flaviviruses. The mAbs were assayed against the DENV-1 (BR/90), -2 (ICC 266), -3 (290-02) and -4 (TVP 360) isolates. Several recognition patterns were identified: group-specific (DENV-1, -2, -3 and -4), subcomplex-specific (DENV-1, -3 and -4, and DENV-2 and -3) and serotype-specific (DENV-2 or -3). Eight mAbs recognized the four DENV serotypes. One mAb reacted with DENV serotypes 1, 3 and 4, and one reacted with serotypes 2 and 3. Three mAbs reacted specifically to serotype 2 and nine reacted to serotype 3 (Table 1 and Figure 2). All mAbs showed the same characteristic staining pattern in IFA in C6/36 infected cells, with a strong perinuclear stain, as illustrated in the reaction with mAb D3 424/8G (Figure 2).

**Figure 3:**
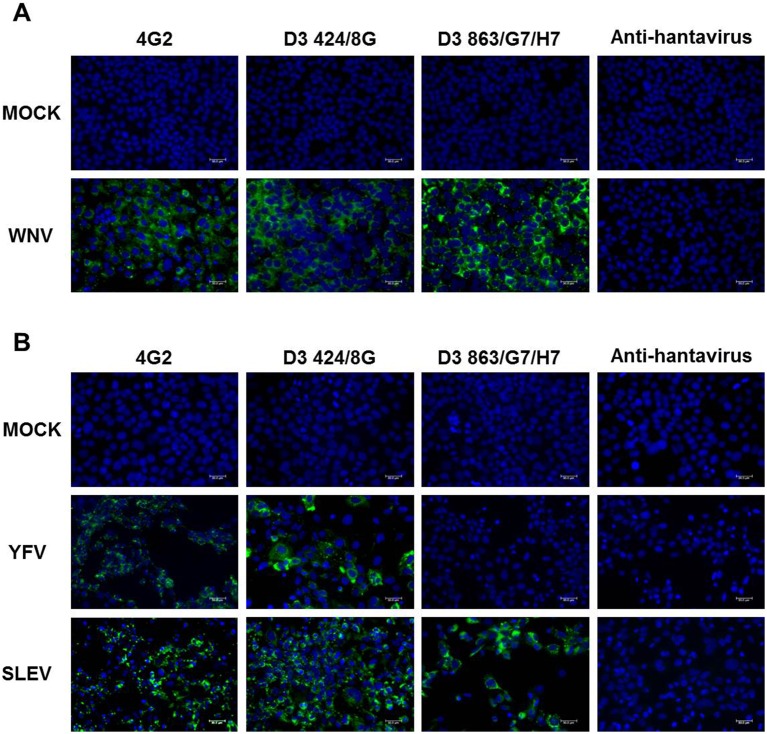
Cross-reactivity of mAbs D3 424/8G and D3 863/G7/H7 against WNV, SLEV and YFV. Vero E6 cells were infected with WNV (A), whereas Huh7.5 cells were infected with YFV and SLEV (B). Cells were fixed in methanol:acetone and stained with mAbs, followed by Alexa-Fluor 488-conjugated anti-mouse immunoglobulin. Monoclonal antibody 4G2 and a non-correlated anti-hantavirus mAb (572/7A) were used as positive and negative controls, respectively. Images were obtained with a Leica AF6000 Modular System. Scale bars are 30 µm.

Moreover, the reactivity of the mAbs was also tested against YFV 17DD, the SLEV 78V6507 isolate, the WNV E/7229/06 isolate and the VEEV TC38 strain. D3 424/8G recognized SLEV, WNV and YFV and did not cross-react with the alphavirus VEEV, suggesting that it is flavivirus-specific (Figure 3 and Table 2). Monoclonal antibodies directed against prM from DENV, D3 443/H12/H6, D3 457/H7/H2, D3 863/G7/H7 and D3 868/G7/H10 recognized the four DENV serotypes, SLEV and WNV but did not react against YFV or VEEV (Table 2). The positive control 4G2 reacted with all dengue serotypes (Figure 2) and other flaviviruses (Figure 3). As expected, anti-hantavirus mAb (572/7A) did not react with any of the viruses tested (Figures 2 and 3).

**Table 2:** Cross-reactivity of anti-dengue virus monoclonal antibodies against YFV, SLEV, WNV and VEEV.

mAb	Reactivity against			
	YFV	SLEV	WNV	VEEV
**D1 463/G6/H2**	−	−	−	−
**D1 695/12C/2H**	−	−	−	−
**D1 606/A12/B9**	−	−	−	−
**D2 646/9G**	−	−	−	−
**D2 658/9A**	−	−	−	−
**D2 332/2D**	−	−	−	−
**D3 342/5G/G8**	−	−	−	−
**D3 388/4A/G6**	−	−	−	−
**D3 444/4G/H3**	−	−	−	−
**D3 389/F4/H10**	−	−	−	−
**D3 441/D1/H2**	−	−	−	−
**D3 290/4C/G9**	−	−	−	−
**D3 341/H9/F10**	−	−	−	−
**D3 344/H1**	−	−	−	−
**D3 442/4E/G8**	−	−	−	−
**D3 242/F1/H2**	−	−	−	−
**D3 424/8G**	+	+	+	−
**D3 863/G7/H7**	−	+	+	−
**D3 457/H7/H2**	−	+	+	−
**D3 443/H12/H6**	−	+	+	−
**D3 868/G7/H10**	−	+	+	−
**D3 63/F2/G7**	−	−	−	−

−: negative;

+: positive.

**Figure 4:**
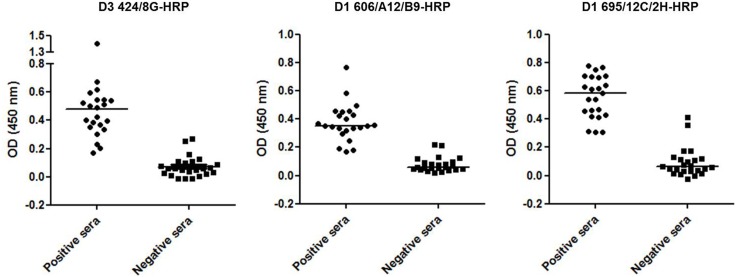
Application of antibodies to the development of MAC-ELISA. HRP-conjugated D3 424/8G, D1 606/A12/B9 and D1 695/12C/2H mAbs were used in an in-house MAC-ELISA assay to detect anti-dengue virus IgM in the sera of infected (N = 22) and non-infected patients (N = 24).

Finally, mAbs D3 424/8G, D1 606/A12/B9 and D1 695/12C/2H were successfully conjugated to HRP for use in diagnostic assays. The three monoclonal antibodies were used to detect dengue virus antigen in human serum samples using an in-house MAC-ELISA (Figure 4). These results are consistent with those from the commercially available PanBio IgM capture assay kit. This method could thus be used to differentiate between negative and positive samples.

## Discussion

Dengue is hyperendemic to tropical and subtropical regions of the world. In Brazil, more than seven million dengue cases have been confirmed since 1986, causing more than two thousand deaths [45]. The co-circulation of the four DENV serotypes and the wide distribution of the mosquito vector *Aedes aegypti* are most likely responsible for the increased incidence and distribution of dengue. Severe clinical manifestations have also increased in recent years, suggesting that dengue should remain a public health priority in Brazil [46]. Therefore, early and accurate diagnosis is essential to reducing morbidity and mortality related to dengue. Commercial kits for dengue diagnosis must be imported at great expense to the Brazilian Ministry of Health.

In this report, we describe the production and characterization of 22 mAbs against Brazilian DENV from the clinical isolates of DENV serotypes 1 (BR-01/MR), 2 (BR/01-01) or 3 (BR 290-02). All of the mAbs showed the same characteristic staining pattern in IFA, with a strong perinuclear stain tending to spread throughout the cytoplasm in fluorescent granules. This observation is consistent with the distribution of DENV-2 proteins observed by Cardiff et al. (1973) [47], who observed an intense perinuclear fluorescence radiating into the cytoplasm in a granular pattern of decreasing intensity. Henchal et al. (1982) found the same fluorescence pattern with monoclonal antibodies after infecting LLC-MK2 cells with a different flavivirus [29]. Both structural and non-structural proteins may localize in the perinuclear region before virus release, causing intense perinuclear staining in this region, whereas cytoplasmic fluorescence is associated with virion antigens [47].

Western blot and IFA analyses showed that most of the mAbs produced in this study are specific to the E or the prM proteins of DENV. The mice were immunized with the virion particle, and DENV does not replicate well in immunocompetent mice [48], [49]. Usually, structural proteins are the major antigens that stimulate the immune response [50]. In humans, proteins E and prM, together with the non-structural protein 1 (NS1), are the major targets of the antibody response during DENV infection, especially in primary infection [51], [52].

The envelope protein from DENV-2, recognized by both the mAbs D2 332/2D and the positive control 4G2, appeared as two bands in the WB, which may represent different glycosylation patterns of the E protein [53]. The other antibodies raised against DENV-2 (D2 646/9G and D2 658/9A) did not recognize DENV antigens in the IFA and WB assays. These mAbs may recognize a non-structural protein or a conformational epitope in structural proteins. D3 342/5G/G8, D3 290/4C/G9, D3 341/H9/F10, D3 424/G8 and the positive control, 4G2, recognized bands that may represent dimeric and trimeric forms of the DENV E protein, suggesting the presence of epitopes exposed in the three different forms of dengue E protein [54]. Also, variability on band intensities observed in WB could be due to differences in mAbs concentration since unpurified supernatants have been used in the assays.

Additionally, mAb D3 63/F2/G7 recognized DENV-3-E protein only in the WB assay and not recombinant DENV-3 E Δ_101_ protein. Since the recombinant E protein lacks the last 101 amino acids corresponding to the juxtamembrane steam region and the transmembrane anchor [55], the mAb D3 63/F2/G7 would recognize an epitope located at this domain. Also, this finding is consistent with reports describing flavivirus mAbs directed to cryptic epitopes of E protein [25], [56], [57]. Some epitopes are readily available on the surface of mature DENV, whereas others are partially or completely inaccessible. Denaturation of viral particles renders the cryptic epitopes accessible, allowing the antibodies to bind. Stiasny et al. (2006) isolated cross-reactive antibodies directed to a cluster of epitopes that are partially occluded in the cage-like assembly of E proteins located at the surface of infectious virions of tick-borne encephalitis virus (TBEV) [56]. Austin et al. (2012) and Chan et al. (2012), respectively, reported the isolation of a mAb able to bind to cryptic epitopes located at a CC′ loop epitope on domain III (DIII) of the E protein from two different DENV-1 genotypes and the isolation of a human prM-specific antibody that bound a cryptic epitope located in the DI/DII junction on the DENV E glycoprotein [25], [57].

Some flavivirus epitopes are also shared by other viruses in this family. mAbs raised against JEV E protein cross-reacted against Murray Valley encephalitis (MVE), WNV, SLE and DENV-1 and -2 [58]. Aside from the four dengue serotypes, the monoclonal antibody D3 424/8G generated in this study recognizes WNV, SLE and YFV. It thus represents a candidate for the development of flavivirus diagnostic assays. Henchal et al. (1982) developed antibodies that recognize flavivirus group-specific viruses, the four DENV serotypes, YFV, WNV, SLEV, Ntaya virus (NTA), Langat virus (LGT), Kunjin virus (Kun), Japanese encephalitis virus (JEV), Ilheus virus (ILH), Banzi virus (BAN) and Zika virus [29]. Additionally, dengue complex-specific (four DENV serotypes), subcomplex-specific (DENV-1, DENV-3) and serotype-specific mAbs were raised [29].

Serological diagnosis of flavivirus infections is difficult due to the extensive antigenic cross-reactivity among these viruses [58]. Well-characterized dengue-specific mAbs are thus powerful tools. To evaluate the applicability of mAbs to the development of immunoassays for dengue virus detection, dengue group-specific anti-E D1 695/12C/2H, anti-prM D1 606/A12/B9 and a flavivirus-specific anti-E D3 424/8G were used in an in-house IgM-capture assay. HRP-conjugated mAbs were successfully used in an anti-IgM capture immunoassay for dengue [59]. Additionally, serotype-specific mAbs (Table 1) could be valuable in an ELISA for serotyping dengue infections [60]. Furthermore, murine mAbs have also been used to detect DENV by immunohistochemistry [31], indicating another possible use for dengue mAbs conjugated to HRP. Finally, mAbs could also be labeled with other molecules such as fluorochromes or colloidal gold for routine dengue diagnosis in other formats.

In conclusion, twenty-two mAbs raised against Brazilian dengue virus isolates, including flavivirus cross-reactive, dengue-group specific, dengue subcomplex-specific and dengue serotype-specific mAbs, may be useful for the development of immunoassays such as ELISA, immunochromatographic assays, dot-blot assays and immunofluorescence assays [10], [14], [44]. To our knowledge, these are the first mAbs against dengue virus isolates circulating in Brazil to be developed and characterized. These mAbs thus have the potential to increase the specificity of dengue diagnosis in this region.

## Supporting Information

Figure S1 **Western blot analysis of DENV-2 mAbs reactivity against Domain III of E protein expressed in** ***E. coli*** **.** Recombinant Domain III of E protein was subjected to 15% SDS-PAGE and electroblotted onto nitrocellulose membranes. Domain III (∼12 kDa) were stained with the mAbs D2 332/2D, D2 658/9A and D2 646/9G, followed by anti-mouse IgG conjugated to alkaline phosphatase. A mouse polyclonal anti-DENV-2 serum was used as positive control.
(TIF)

Figure S2 **Monoclonal reactivities on immunofluorescence assay (IFA) against recombinant DENV-3 E Δ_101_ protein expressed on** ***Drosophila*** **S2 cells.** Indirect immunofluorescence of *Drosophila* S2 cells expressing or not (Mock) recombinant DENV-3 E Δ_101_ protein with mAbs D3 388/4A/G6, D3 344/H1 and D3 63/F2/G7. Monoclonal antibody 4G2 and a non-correlated anti-hantavirus mAb (clone 572/7A) were used as positive and negative controls, respectively. Images were produced in a Leica AF6000 Modular System. Scale bars are 75 µm.
(TIF)

Table S1 **Reactivity with recombinant DENV-3 E Δ_101_ protein expressed on** ***Drosophila*** **S2 cells against twelve mAbs anti-DENV-3/E.**
(PDF)
