# Supplementary figures and images for: Development, Characterization and Application of Monoclonal Antibodies against Brazilian Dengue Virus Isolates
Source: PLoS One. 2014 Nov 20;9(11):e110620. doi: 10.1371/journal.pone.0110620 (PMC4239016; doi:10.1371/journal.pone.0110620)

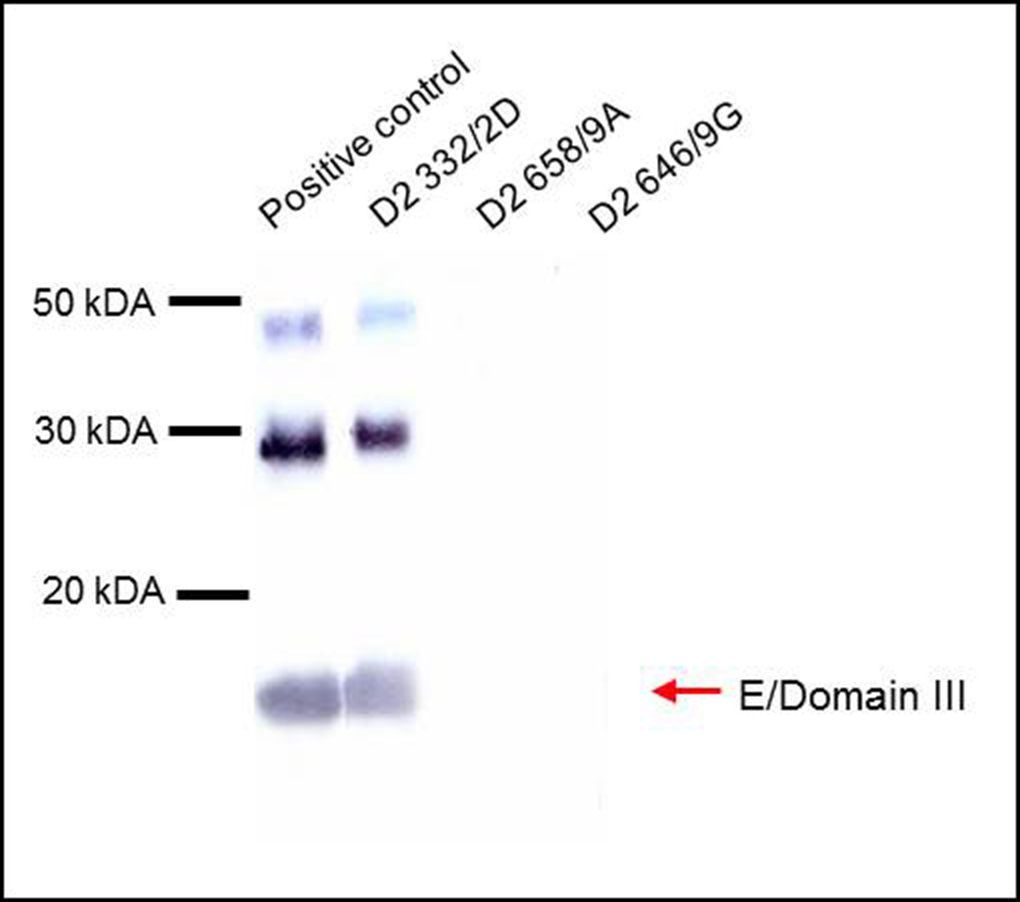

Supplement: Figure S1 — Western blot analysis of DENV-2 mAbs reactivity against Domain III of E protein expressed in E. coli . Recombinant Domain III of E protein was subjected to 15% SDS-PAGE and electroblotted onto nitrocellulose membranes. Domain III (∼12 kDa) were stained with the mAbs D2 332/2D, D2 658/9A and D2 646/9G, followed by anti-mouse IgG conjugated to alkaline phosphatase. A mouse polyclonal anti-DENV-2 serum was used as positive control. (TIF) [file pone.0110620.s001.tif]

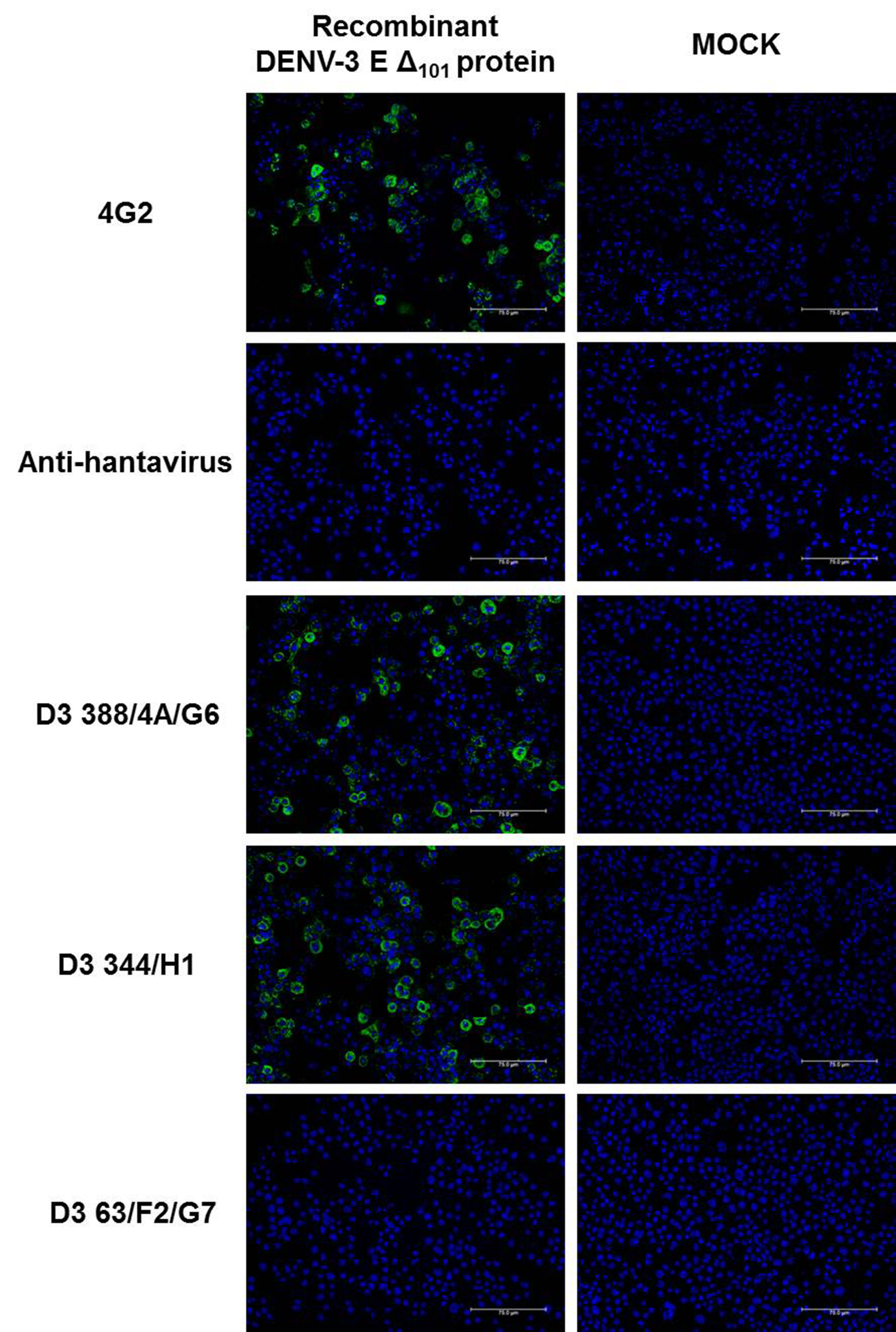

Supplement: Figure S2 — Monoclonal reactivities on immunofluorescence assay (IFA) against recombinant DENV-3 E Δ101 protein expressed on Drosophila S2 cells. Indirect immunofluorescence of Drosophila S2 cells expressing or not (Mock) recombinant DENV-3 E Δ101 protein with mAbs D3 388/4A/G6, D3 344/H1 and D3 63/F2/G7. Monoclonal antibody 4G2 and a non-correlated anti-hantavirus mAb (clone 572/7A) were used as positive and negative controls, respectively. Images were produced in a Leica AF6000 Modular System. Scale bars are 75 µm. (TIF) [file pone.0110620.s002.tif]
