# Supplementary material for: Development, Characterization and Application of Monoclonal Antibodies against Brazilian Dengue Virus Isolates
Source: PLoS One. 2014 Nov 20;9(11):e110620. doi: 10.1371/journal.pone.0110620 (PMC4239016; doi:10.1371/journal.pone.0110620)
Supplement: Table S1 — Reactivity with recombinant DENV-3 E Δ101 protein expressed on Drosophila S2 cells against twelve mAbs anti-DENV-3/E. (PDF) [file pone.0110620.s003.pdf]

mAb	Reactivity with recombinant DENV-3 E Δ_{101} protein
D3 342/5G/G8	+
D3 388/4A/G6	+
D3 444/4G/H3	+
D3 389/F4/H10	+
D3 441/D1/H2	+
D3 290/4C/G9	+
D3 341/H9/F10	+
D3 344/H1	+
D3 442/4E/G8	+
D3 242/F1/H2	+
D3 424/8G	+
D3 863/G7/H7	-
D3 457/H7/H2	-
D3 443/H12/H6	-
D3 868/G7/H10	-
D3 63/F2/G7	-
